# Supplementary material for: Dynalign II: common secondary structure prediction for RNA homologs with domain insertions
Source: Nucleic Acids Res. 2014 Nov 21;42(22):13939–48. doi: 10.1093/nar/gku1172 (PMC4267632; doi:10.1093/nar/gku1172)
Supplement: SUPPLEMENTARY DATA [file supp_gku1172_nar-02021-z-2014-File012.zip › manual/RNA_class/html/globals.html]

RNAstructure Classes: Class Members

- Main Page
- Classes
- Files

- File List
- File Members

- All
- Functions
- Variables
- Defines

Here is a list of all file members with links to the files they belong to:

- COMPOUNDMESSAGELENGTH
  : TwoRNA.h- DYNALIGNOBJECT\_H
    : Dynalign\_object.h- epsilon
      : RNA.cpp- errmsg()
        : RNA.cpp- HYBRIDRNA\_H
          : HybridRNA.h- main()
            : Dynalign\_class.cpp
            , RNA\_class.cpp
            , OligoWalk\_class.cpp
            , HybridRNA\_class.cpp
            , DynalignSMP\_class.cpp- OLIGOWALKOBJECT\_H
              : Oligowalk\_object.h- RNA\_H
                : RNA.h- THERMODYNAMICS\_H
                  : thermodynamics.h- TOLERANCE
                    : thermodynamics.h- TWORNA\_H
                      : TwoRNA.h

---

Generated on Tue Jul 30 10:52:29 2013 for RNAstructure Classes by 
 1.5.7.1
